# A systematic high-throughput phenotyping assay for sugarcane stalk quality characterization by near-infrared spectroscopy

**DOI:** 10.1186/s13007-021-00777-8

**Published:** 2021-07-13

**Authors:** Maoyao Wang, Xinru Li, Yinjuan Shen, Muhammad Adnan, Le Mao, Pan Lu, Qian Hu, Fuhong Jiang, Muhammad Tahir Khan, Zuhu Deng, Baoshan Chen, Jiangfeng Huang, Muqing Zhang

**Affiliations:** 1 Guangxi Key Laboratory of Sugarcane Biology, Sugar Industry Collaborative Innovation Center, State Key Laboratory for Conservation and Utilization of Subtropical Agro-Bioresources, College of Agriculture, Guangxi University, Nanning, 530004 Guangxi China; 2 National Engineering Technology Research Center of Sugarcane, Fujian Agriculture and Forestry University, Fuzhou, 350002 Fujian China; 3 Sugarcane Biotechnology Group, Nuclear Institute of Agriculture (NIA), Tandojam, Pakistan

**Keywords:** Sugarcane, Culm sugar content, Biomass, NIRS, HPAEC

## Abstract

**Background:**

Sugarcane (*Saccharum officinarum* L.) is an economically important crop with stalks as the harvest organs. Improvement in stalk quality is deemed a promising strategy for enhancing sugarcane production. However, the lack of efficient approaches for systematic evaluation of sugarcane germplasm largely limits improvements in stalk quality. This study is designed to develop a systematic near-infrared spectroscopy (NIRS) assay for high-throughput phenotyping of sugarcane stalk quality, thereby providing a feasible solution for precise evaluation of sugarcane germplasm.

**Results:**

A total of 628 sugarcane accessions harvested at different growth stages before and after maturity were employed to take a high-throughput assay to determine sugarcane stalk quality. Based on high-performance anion chromatography (HPAEC-PAD), large variations in sugarcane stalk quality were detected in terms of biomass composition and the corresponding fundamental ratios. Online and offline NIRS modeling strategies were applied for multiple purpose calibration with partial least square (PLS) regression analysis. Consequently, 25 equations were generated with excellent determination coefficients (*R*^*2*^) and ratio performance deviation (RPD) values. Notably, for some observations, RPD values as high as 6.3 were observed, which indicated their exceptional performance and predictive capability.

**Conclusions:**

This study provides a feasible method for consistent and high-throughput assessment of stalk quality in terms of moisture, soluble sugar, insoluble residue and the corresponding fundamental ratios. The proposed method permits large-scale screening of optimal sugarcane germplasm for sugarcane stalk quality breeding and beyond.

**Supplementary Information:**

The online version contains supplementary material available at 10.1186/s13007-021-00777-8.

## Background

Sugarcane (*Saccharum officinarum* L.) is a perennial C4 crop cultivated worldwide in subtropical and tropical zones. It is one of the most important industrial crops for sugar and ethanol production [1]. Moreover, sugarcane is an exceptionally productive commodity that is locally processed into value-added products and contributes to the economic welfare of cultivating areas.

Sugarcane stalk quality plays a decisive role in the profitability of this crop. Sugar is the primary industrial product from sugarcane stalks. Strenuous efforts have been expended in recent decades to obtain more sugar from cane stalks. However, because of the complicated carbon partitioning and sugar accumulation mechanisms, limited achievements have been realized [2–4]. Recently, advances in genomic tools and the decreasing costs of sequencing have enabled plant breeders to pursue large-scale precision breeding [5, 6]. In addition to these advances, the use of high-throughput phenotyping is anticipated to play a significant role in accelerating improvement in crop genetics [7, 8].

Sugarcane stalk consists of water, sugar, and fiber. These three significant components result in dynamic variations in sugarcane stalks among different genotypes, growth periods, and meteorological conditions [9]. The dry mass of cane stalks is composed of sugar and fiber, so the sugar concentration is therefore influenced by partitioning of carbon between the two [10]. Payment for sugarcane is closely related to the sucrose concentration in fresh stalks, which is determined by the concentration of stalk sucrose on a dry weight basis (g sucrose g/g DW) and moisture content (g water g/g FW). Moreover, sugar composition and the ratios among different sugar forms also exhibit variation between different genotypes and within a single genotype during sugarcane ripening [11, 12]. For instance, with the rapid accumulation of sucrose, the content of reducing sugars (glucose and fructose) gradually decreases as sugarcane matures. Hence, the ratio of reducing sugars to sucrose is usually used to evaluate the degree of sugarcane maturity [13]. The precise analysis of these compounds in a high-throughput method may facilitate large-scale accurate phenotyping of sugarcane stem quality.

Near-infrared spectroscopy (NIRS) is highly efficient and has been applied for high-throughput screening to predict the properties and compositions of large numbers of samples [14], especially for phenotyping and genomic selection in crop breeding [15, 16]. It has been used for quality trait (such as juice soluble solids content, i.e., Brix, juice pH, firmness and water content) phenotyping in tomatoes [17]; estimation of sucrose, glucose, and fructose in sweet sorghum juice [18]; phenotyping of malt extract and protein content in barley [19]; assessment of amino acid concentrations for quantitative trait locus (QTL) analysis in soybean [20]; quantitative monitoring of sucrose, reducing sugars and total sugar dynamics for phenotyping of water-deficit stress tolerance in rice [21]; prediction of silage quality traits for QTL mapping in maize [22]; and herbage quality trait analysis [23]. In addition, NIRS has also been used to determine chemical compounds in sugarcane, which is used for analysis of phosphorus in leaves [24], estimation of mineral content under saline conditions [25], and estimation of cell wall components in stalks [26]. Some studies have also involved the use of NIRS calibration for sugar concentration in juices in terms of Brix or pol values [26, 27] or commercial cane sugar contents [28]. However, little research has systematically explored NIRS assays for high-throughput characterization of sugarcane stalk quality with the compounds described above.

In this work, hundreds of samples were collected from various genotypes at different growth stages. Stalk quality was assessed by quantitatively analyzing the chemical composition and the corresponding ratio values in sugarcane stalk tissues via a high-performance anion chromatography (HPAEC-PAD) assay. Considerable variations in stalk quality were observed within these collections, allowing for consistent offline and online NIRS calibration in sugarcane. Therefore, this study provided systematic and multiple options-based assays for high-throughput screening of stalk quality, allowing for large-scale phenotyping of sugarcane germplasm during precision breeding.

## Results and discussion

### Precise sugar content determination in sugarcane stalks

**Fig. 1:**
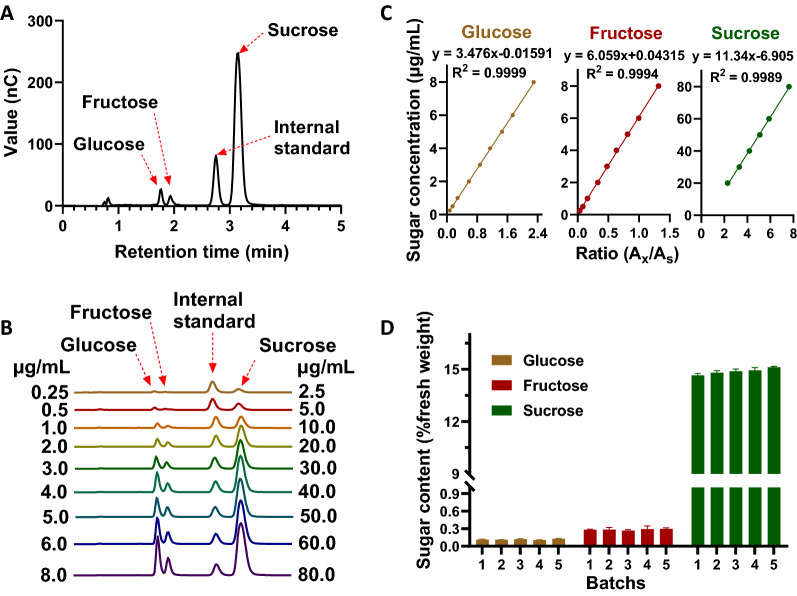
High-performance anion chromatography assay for sugar determination in sugarcane. **A** Chromatogram of sugar determination in sugarcane; **B** chromatogram of standard mixtures at different concentrations; **C** standard curves; **D** sugar determination in different batches of sugarcane

HPAEC-PAD assay was performed to detect sugar content in sugarcane stalks, and the standard internal method was adapted for quantitative analyses. In this assay system, all target compounds (glucose, fructose, and sucrose) and the internal standard (lactose) were separated entirely within 3.5 min (Fig. 1A). Therefore, the method allowed for rapid analysis of sugar content in sugarcane stalks. The reducing sugar (glucose and fructose) content should be much lower than that of sucrose in mature stems of sugarcane [13, 29]. To obtain more accurate equations for quantitative analysis, a gap of 10 times the difference in these concentrations was set between the sugars in the gradient mixtures used to prepare standard curves. Expressly, the standard mixture for glucose and fructose was set to range from 0.25 to 8.0 μg/mL, while sucrose ranged from 2.5 to 80 μg/mL (Fig. 1B). As a result, high *R*^*2*^ values were observed for the standard curves (glucose, fructose, and sucrose) of each sugar (Fig. 1C), which indicated the reliability of the quantitative analysis. In addition, to determine if batch effects were present in these laboratory assays, the same sample was used for quantitative analysis of sugar content in different experimental batches. As shown in Fig. 1D, no significant differences were observed between batches. Thus, the results indicated that there was no batch effect in this quantitative assay, suggesting that all samples tested in the individual experimental batches could be combined for integrative analysis. Moreover, to check whether sugar was lost during the sample drying process, a comparative analysis was carried out to determine the sucrose content in fresh and dried samples. No significant differences were detected between them (Additional file 1: Figure S1). These results indicated the establishment of a rapid and stable HPAEC assay that allows for accurate analyses of sugar content in sugarcane stalks.

### Diverse biomass composition in collected sugarcane stalks

**Fig. 2:**
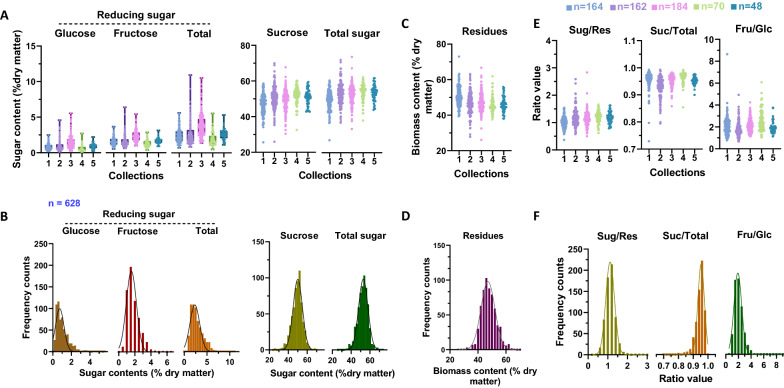
Variations in dry biomass composition in sugarcane stalks. Sugar mass content (**A**) and frequency distribution (**B**) in sugarcane stalks; insoluble residue content (**C**) and frequency distribution (**D**) in sugarcane stalks; ratio (**E**) between biomass composition and its frequency distribution (**F**). Various genotypes of sugarcane were collected at five different times, and the numbers for the collections were 164, 162, 184, 70 and 48. Samples in different collections were merged together (n = 628) to calculate the distribution frequency of biomass component composition in B, D and F. Sug/Res, total soluble sugar/residues; Suc/Total, sucrose/total soluble sugar; Fru/Glc, ratio of fructose/glucose in soluble sugar

Biomass composition, especially the sugar content in cane stalks, is critical for classification of quality. To obtain samples with sufficient variability in biomass composition, sugarcane stalks of different genotypes were collected once per month from November 2018 to March 2019. The sugar mass content (g/g, % dry weight) of the ground dry samples was determined by the HPAEC-PAD assay described above. Due to genotype diversity, large variations were detected in each collection (Fig. 2A). Samples in collection 2 showed the highest diversity for reducing sugars, sucrose, and total soluble sugars. Notably, continuous increases in sucrose and total soluble sugar contents (g/g, % dry weight) were observed from collection 1–5 (Fig. 2A), which was due to the increasing maturation of the sugarcane stalks between November 2018 and March 2019. The different collections were combined to obtain a large sample set for NIRS calibration, as shown in Fig. 2B. The integrated sample set exhibited a more comprehensive range of variation and better normal distribution compared to the constituents. In detail, the reducing sugar content (g/g, % dry weight) ranged from 0.48 to 10.96 (average value at 2.87), sucrose ranged from 25.61 to 69.92, and total soluble sugar content ranged from 27.02 to 73.88 (Fig. 2B). In sugarcane stems, soluble sugars and insoluble residues are central dry mass components formed by photosynthesis. The insoluble residue was calculated by deducting the total soluble sugar content from the dry biomass. Even though the contents of insoluble residue (g/g, % dry weight) in the collected sugarcane samples gradually decreased from collection 1–5 (Fig. 2C), a normal distribution ranging from 26.12 to 72.98 was observed in the combined sample set (Fig. 2D).

Generally, the proportion of chemical components in the sugarcane stalk is considered an important index for evaluating quality [30]. For example, the ratio between sugar and residues (Sug/Res) is closely related to the carbon partitioning patterns that primarily determine clean sugar production in sugarcane stalks. In comparison, the sucrose proportion in total soluble sugar (Suc/Total) is recognized as the critical index for judging juice purity. The ratio between fructose/glucose (reducing sugars) relates to the physiological development of sugarcane [30, 31]. The values of these fundamental ratios described above were calculated to allow systematic characterizations of sugarcane stalk quality. As expected, considerable variation in these ratios was observed in the collected sugarcane population (Fig. 2E, F). Notably, the Sug/Res value ranged from 0.37 to 2.83, and a high coefficient of variation (CV) value was observed (0.23), suggesting a broad diversity of carbon partitioning patterns in the sugarcane population. In contrast, the Suc/Total value showed limited variation because most of the collected samples had almost matured during the study period.

For commercial sugarcane production, sugarcane stalk quality is determined by sucrose concentration on a fresh weight basis (g sucrose g/g fresh weight). However, in fresh sugarcane stalks, the sugar concentration is not only related to the mass content of sugar in the dry matter but also depends on water content. The accumulation of % soluble sugars is reportedly associated with a concomitant decrease in moisture content [32]. An increase in sucrose content expressed in terms of fresh mass may occur even without the deposition of additional sucrose when culms become dehydrated due to low levels of soil moisture. Thus, sugarcane ripening expressed as % increase in sucrose content does not necessarily depict sucrose content [33]. Therefore, in sugarcane, the high sugar mass content (g/g, % dry weight) and low moisture content (g/g, % fresh weight) could be considered optimal criteria for judging sugar production.

**Fig. 3:**
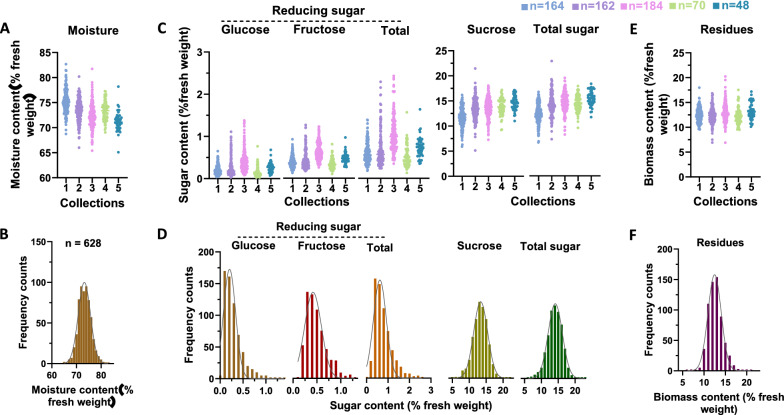
Variations in fresh biomass composition in sugarcane stalks. Moisture content (**A**) and frequency distribution (**B**) in sugarcane stalks; sugar content (**C**) and frequency distribution (**D**) in sugarcane stalks; insoluble residue content (**E**) and frequency distribution (**F**) in sugarcane stalks. Various genotypes of sugarcane were collected at five different times, and the numbers for each collection were 164, 162, 184, 70 and 48. Samples in different collections were merged together (n = 628) to calculate the distribution frequency of biomass component composition in B, D and F

This study also determined biomass concentrations in fresh sugarcane stalks according to their dry biomass and moisture content. Owing to the classic drying water loss method [34], moisture content diversity was detected in the collected sugarcane population (Fig. 3A, B). The water content of sugarcane decreased significantly from collection 1–5, which may be related to the gradual loss of water in the later stages of sugarcane growth (Fig. 3A). In contrast, with decreasing water content in sugarcane stalks, the sugar concentration (g/g, % fresh weight), mainly sucrose and total soluble sugar concentrations, gradually increased (Fig. 3B). However, the concentrations of the residues (g/g, % fresh weight) seemed to be similar among the different collections (Fig. 3E). As expected, all of these compounds displayed considerable variation and led to a normal distribution in the combined sample set (Fig. 3B, D, F), which indicated the accurate calibration with NIRS.

### NIRS data characterization in collected sugarcane stalks

**Fig. 4:**
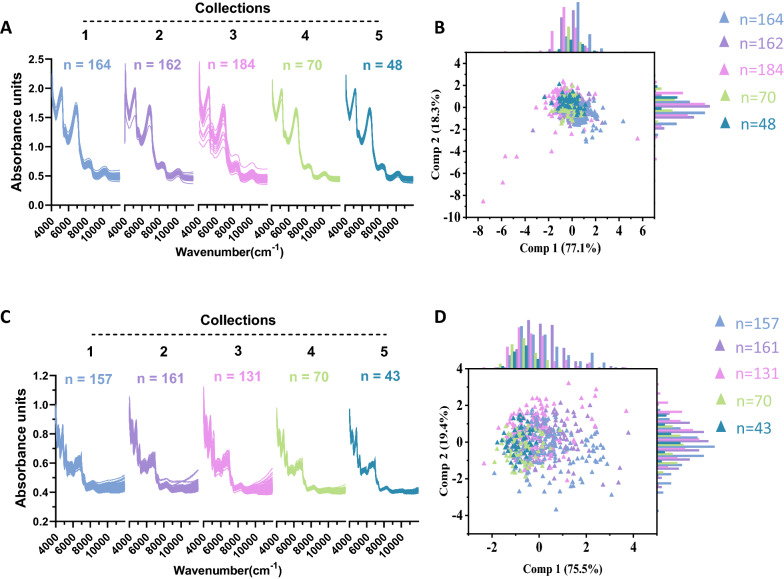
Variations of NIRS absorbance spectra for sugarcane samples. Original spectra of fresh (**A**) and dry samples (**C**); PCA scores of near-infrared spectra for fresh (**B**) and dry samples **D**

DM540-CPS coupled with the MATRIX-F system has been designed explicitly for sugarcane quality control (QC) analysis based on the related calibration model [35]. Sugarcane stalks were shredded and automatically passed to the NIR sensor for collection of spectral data within one minute. Instead of pressing out the cane juice for quantitative analysis, no juice was extracted from the sugarcane stalks. To establish the biomass composition calibration model for online quantitative analysis, near-infrared spectra for fresh sugarcane stalks in each collection were recorded on this system. As a result, broad diversity was detected among sugarcane samples (Fig. 4A). PCA was carried out to characterize the structure of the combined spectral population [36], as shown in Fig. 4B; no significant discrimination could be detected among these spectra from different collections. The continuous distribution of the combined spectral population further indicated that these samples could be integrated into a global NIRS calibration population. In addition, during the PCA, the global distance (GH) between each spectrum was calculated, and the GH outliers were eliminated from the population during further NIRS modeling [37, 38].

As a comparison, an offline near-infrared spectroscopy data scanning assay was applied to perform offline NIRS calibration. Dry ground samples from different collections were scanned offline by a MATRIX-F equipped with a Q413 sensor. It was apparent that the spectrum of the ground dry sample was different from that of the fresh sample (Fig. 4C) but showed a pattern similar with that of a previous report on dry samples in sugarcane and some other species [39–42], which can be attributed to water loss [42, 43]. PCA results showed that the spectrum of the dry sample exhibited much higher variation (Fig. 4D), indicating that the offline assay spectra would be more conducive for NIRS modeling.

### Determination of calibration and validation sets

A total of 562 samples in the combined sets were obtained for offline NIRS modeling. One fifth of the samples was randomly selected into the validation sets, while the remaining 449 samples formed the calibration sets. A descriptive statistical analysis was conducted to compare the calibration and validation sets in terms of the minimum (Min), maximum (Max), mean, standard deviation (SD), and coefficient of variation (CV) values (Table 1). Similarly, 628 samples were used for online NIRS modeling either for biomass composition content in dry weight (g/g, % dry weight) or in fresh sugarcane stalks (g/g, % fresh weight). Before NIRS modeling, 502 samples were randomly placed into the calibration sets, and the remaining 126 were included in the validation sets (Table 1). As shown in Table 1, all samples in the calibration and validation sets showed comparable statistical distributions, allowing reliable NIRS modeling.

**Table 1:** Calibration and validation sets for biomass components in sugarcane stalks

	Calibration						Validation					
	N	Min	Max	Mean	SD	CV	N	Min	Max	Mean	SD	CV
Dry weight (offline)												
Sugars												
Glucose	449	0.10	4.76	0.98	0.69	0.71	113	0.22	5.55	1.29	0.99	0.77
Fructose	449	0.38	4.49	1.72	0.72	0.42	113	0.77	6.38	2.00	0.96	0.48
Reducing sugar	449	0.48	8.33	2.69	1.36	0.50	113	1.09	10.96	3.29	1.91	0.58
Sucrose	449	25.61	69.92	49.68	5.69	0.11	113	25.93	60.35	48.51	5.98	0.12
Total sugar	499	27.02	73.88	52.37	5.64	0.11	113	36.89	63.16	51.80	5.34	0.10
Residues	449	27.83	72.98	47.93	5.46	0.11	113	26.12	63.11	47.83	6.13	0.13
Ratio												
Sug/res	449	0.37	2.83	1.13	0.27	0.24	113	0.58	1.71	1.10	0.23	0.21
Suc/total	449	0.81	0.99	0.95	0.03	0.03	113	0.70	0.98	0.94	0.04	0.05
Fru/glc	449	0.67	8.65	2.12	0.78	0.37	113	0.74	4.03	1.89	0.65	0.34
Dry weight (online)												
Sugars												
Glucose	502	0.10	5.12	1.04	0.72	0.69	126	0.23	5.55	1.14	0.94	0.82
Fructose	502	0.38	5.42	1.79	0.74	0.41	126	0.74	6.38	1.92	0.94	0.49
Reducing sugar	502	0.48	10.54	2.83	1.41	0.50	126	1.01	10.96	3.06	1.82	0.60
Sucrose	502	30.45	69.92	49.77	5.49	0.11	126	25.61	60.26	48.08	6.37	0.13
Total sugar	502	33.33	73.88	52.60	5.41	0.10	126	27.02	63.43	51.14	5.83	0.11
Residues	502	26.12	66.67	47.40	5.41	0.11	126	36.57	72.98	48.86	5.83	0.12
Ratio												
Sug/res	502	0.50	2.83	1.14	0.26	0.23	126	0.37	1.73	1.07	0.24	0.22
Suc/total	502	0.79	0.99	0.95	0.03	0.03	126	0.70	0.98	0.94	0.04	0.04
Fru/glc	502	0.69	8.65	2.07	0.78	0.34	126	0.67	3.97	2.04	0.63	0.31
Fresh weight (online)												
Moisture	502	65.10	81.74	73.30	2.43	0.03	126	65.40	82.68	73.52	3.12	0.04
Sugars												
Glucose	502	0.03	1.34	0.28	0.20	0.71	126	0.06	1.38	0.30	0.24	0.81
Fructose	502	0.10	1.22	0.48	0.20	0.43	126	0.19	1.26	0.50	0.23	0.47
Reducing sugar	502	0.13	2.45	0.76	0.39	0.52	126	0.26	2.40	0.80	0.46	0.58
Sucrose	502	7.29	21.47	13.31	2.05	0.15	126	5.14	17.67	12.79	2.46	0.19
Total sugar	502	7.98	22.86	14.07	2.11	0.15	126	6.66	18.91	13.60	2.46	0.18
Residues	502	6.90	20.24	12.63	1.66	0.13	126	9.50	18.07	12.89	1.84	0.14

N, sample number; Min, minimum value; Max, maximum value; SD, standard deviation; CV, coefficient of variation

### NIRS modeling for biomass compositions in sugarcane stalks

Partial least square (PLS) regression analytical methods packed in OPUS software were performed for NIRS modeling. The selected wavelengths of near-infrared spectroscopy were pretreated with derivative and scatter correction methods before NIRS calibration. Internal cross-validation and external validation were applied to evaluate the calibration equations. During NIRS calibration, the root mean square error of calibration/cross-validation/external validation (RMSEC/RMSECV/RMSEP), coefficient of determination of calibration/cross-validation/external validation (*R*^*2*^/*R*^*2*^*cv*/*R*^*2*^*ev*) and the ratio performance deviation (RPD) were obtained to select optimal equations.

**Fig. 5:**
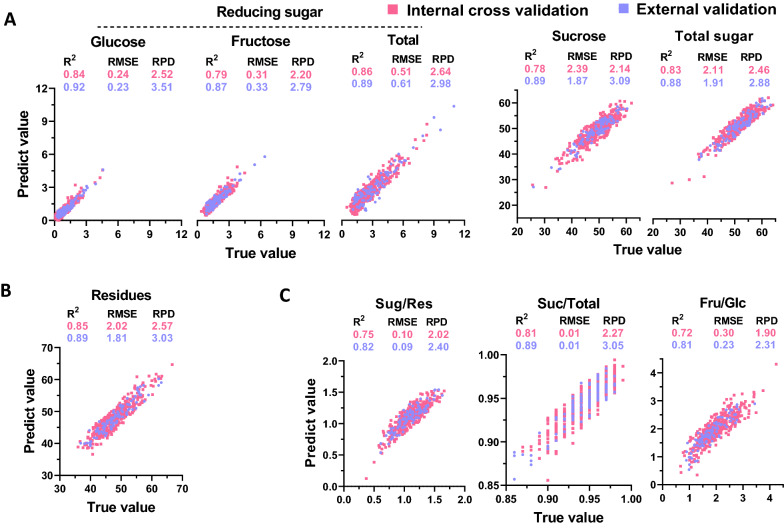
Correlation analysis between predicted and true values for biomass component content (% dry matter) in sugarcane stalks, using offline NIRS calibration. **A** Sugar; **B** insoluble residues; **C** ratio between biomass components. The red and blue dots represent internal cross validation and external validation, respectively. *R*^*2*^, coefficient determination; RMSE, root mean square error; RPD, ratio performance deviation

Due to the absence of water absorption peaks in the near-infrared spectrum, offline NIRS calibration exhibits a great advantage in the determination of dry biomass composition [37, 38, 44]. In this study, the dry ground biomass of sugarcane stalks was used for offline NIRS modeling. The results indicated that all of the equations for sugar, residue content (g/g, % dry weight), and the resulting ratios exhibited high *R*^*2*^ values in calibration. This was especially true for calibration of sugar content (g/g, % dry weight), where the *R*^*2*^ value reached as high as 0.91 (Additional file 1: Table S2). In addition, most of the equations exhibited high *R*^*2*^*cv* and RPD values during internal cross-validation, except for Fru/Glc, which showed a relatively low RPD value of 1.90 (Fig. 5 and Additional file 1: Table S2). Moreover, additional external validation was applied to evaluate the performance of the equations obtained. All of the equations exhibited high linear correlations between predicted and actual values. Glucose (g/g, % dry weight) showed the highest *R*^*2*^*cv* value of 0.92 (Fig. 5 and Additional file 1: Table S2). Notably, all of these equations showed the RPD value much higher than 2.0 during external validation (Fig. 5). Generally, the RPD values (> 2.0) of the equations were attesting to their validity [45–47]; therefore, all of the equations obtained in this study for biomass composition (g/g, % dry weight) exhibited good predictive capabilities in offline NIRS detection systems.

**Fig. 6:**
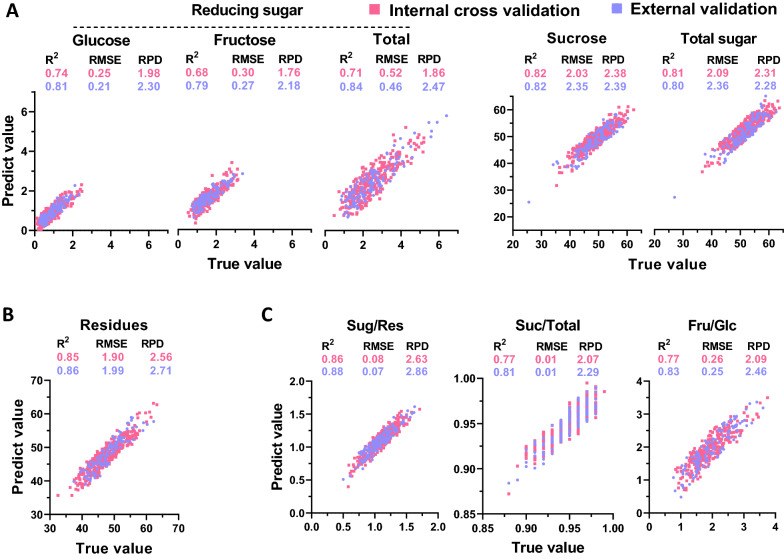
Correlation between predicted and true values for biomass component content (% dry matter) in sugarcane stalks, using online NIRS calibration. **A** Sugar; **B** insoluble residues; and **C** ratios between biomass components. The red and blue dots represent internal cross validation and external validation, respectively. *R*^*2*^, coefficient determination; RMSE, root mean square error; RPD, ratio performance deviation

For comparison, online NIRS modeling was carried out for prediction of dry biomass content (g/g, % dry weight) based on near-infrared spectra collected from fresh sugarcane stalks. The calibration results showed that even though the equations exhibited *R*^*2*^ values that were lower than those of the offline calibration, the results reached a substantially high level of *R*^*2*^ (ranging from 0.83 to 0.91) (Additional file 1: Table S2). Based on cross-validation and external validation data, most of the other equations showed RPD values over 2.0, except for those for reducing sugars (glucose, fructose, and the total, g/g, % dry weight), which showed relatively low *R*^*2*^*cv* values ranging from 0.68 to 0.74 and RPD values ranging from 1.76 to 1.98 (Fig. 6). Notably, the ratio between sugar and residues (Sug/Res) exhibited the best performance in online NIRS calibration. The highest *R*^*2*^, *R*^*2*^*cv*, and *R*^*2*^*ev* values were 0.91, 0.86, and 0.88, respectively (Fig. 6). As the ratio between sugar and residues (Sug/Res) was the key indicator of the carbon partitioning pattern in sugarcane stalks, this NIRS method could provide a reliable high-throughput assay for the large-scale selection of promising germplasms from the sugarcane population.

**Fig. 7:**
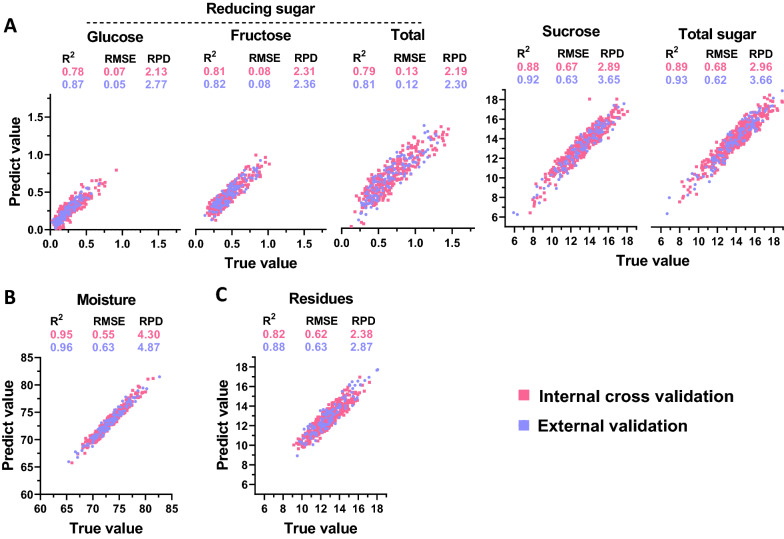
Correlation analysis between the predicted and true values for biomass component content (% fresh weight) in sugarcane stalks upon online NIRS calibration. **A** Sugar; **B** moisture; **C** insoluble residues. The red and blue dots represent internal cross validation and external validation, respectively. *R*^*2*^, coefficient determination; RMSE, root mean square error; RPD, ratio performance deviation

Moreover, near-infrared spectroscopy with fresh sugarcane stalks was applied for online NIRS calibration for prediction of biomass composition (g/g, % fresh weight). For modeling of sugar concentration (g/g, % fresh weight), the equations for sucrose and total soluble sugar exhibited the best performance; *R*^*2*^, *R*^*2*^*cv*, *R*^*2*^*ev*, and RPD values were consistently higher than those for the other equations obtained during calibration and related validations (Fig. 7A and Additional file 1: Table S2). Furthermore, the equations for reducing sugar concentration (g/g, % fresh weight) also exhibited consistently high RPD values, which exceeded 2.0, indicating their excellent predictive capability (Fig. 7A). In particular, the equation for moisture content (g/g, % fresh weight) showed a perfect linear correlation between predicted and actual values, demonstrating reliable and accurate online predictive capability (Fig. 7B). In addition, the residue content (g/g, % fresh weight) also exhibited good predictive performance during calibration and two different kinds of validations, with consistently high *R*^*2*^ values (Fig. 7C).

In comparing online and offline strategies for modeling dry biomass composition, the equations generated by offline calibration showed a higher prediction capacity (Figs. 5, 6). When different sample types were compared during online NIRS modeling, the biomass compositions of fresh samples exhibited much better performances (Figs. 6, 7). Therefore, the data suggested that NIRS strategies could be selected for the sample type to generate the optimal equations for highly accurate predictions.

### Integrative calibration for sugarcane stalk quality

**Fig. 8:**
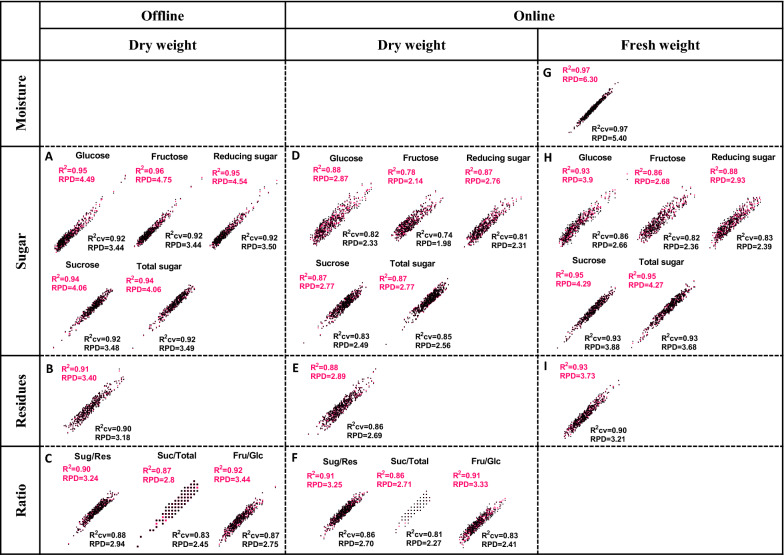
Correlation analysis between the fit (predicted) and true values for biomass component content in sugarcane stalks. Offline NIRS calibration for dry biomass of sugarcane stalks upon sugar content (**A**), residues (**B**) and ratio between them (**C**); **D**–**F** online NIRS calibration for dry biomass of sugarcane stalks upon sugar content (**D**), residues (**E**) and the ratio (**F**); online NIRS calibration for fresh biomass of sugarcane stalks based on moisture content (**G**), sugar content (**H**) and residues (**I**). The red and black colors represent calibration and internal cross validation, respectively

In order to generate a global NIRS calibration, samples in calibration and validation sets were integrated to form a final calibration set. Since more samples were contained in the final calibration set, all of the newly generated equations exhibited much better performance than those described above. In this approach, the average *R*^*2*^ value increased from 0.88 to 0.93 for integrative calibration of offline prediction of dry biomass composition, and the average RPD value increased from 2.3 to 3.2 during cross-validation (Table 2 and Fig. 8A–C). For offline prediction of sugar content (g/g, % dry weight), all of the equations exhibited high *R*^*2*^ and *R*^*2*^*cv* values (over 0.90). The RPD values were higher than 3.0 for calibration and cross-validation (Table 2 and Fig. 8A). Thus, these equations exhibited excellent determinations of sugar contents (g/g, % dry weight) via offline NIRS assay. The performance of online NIRS modeling for dry biomass composition (g/g, % dry weight) did not improve as much as that of offline NIRS modeling due to expansion of the calibration set. However, most of the equations exhibited RPD values over 2.0, permitting reasonable predictions (Table 2 and Fig. 8D–F). Integrative calibration processing enhanced the prediction capacity for online calibration of fresh biomass concentration (g/g, % fresh weight). Notably, apart from reducing sugars (g/g, % fresh weight), which showed *R*^*2*^ and *R*^*2*^*cv* values ranging from 0.82 to 0.93, all of the other equations obtained *R*^*2*^ and *R*^*2*^*cv* values much higher than 0.90 and high RPD values exceeding 3.0 (Table 2 and Fig. 8G–I). Therefore, these newly generated equations could be applied for online quantitative analysis of biomass composition by NIRS assay.

**Table 2:** Integrative calibration statistics for optimized equations generated for prediction of biomass components in sugarcane stalks

	Calibration								Cross validation		
	Rank	N	SCM	Spectrum range (cm^−1^)	Mean	SD	RMSEC	R^2^	RMSECV	R^2^cv	RPD
Dry weight (offline)											
Sugars (% dry weigh)											
Glucose	15	464	FD	3996–11,988	0.96	0.66	0.15	0.95	0.19	0.92	3.44
Fructose	16	418	FD	3996–11,988	1.70	0.75	0.16	0.96	0.22	0.92	3.44
Reducing sugar	15	457	FD	3996–11,988	2.74	1.48	0.33	0.95	0.42	0.92	3.50
Sucrose	10	394	FD + SNV	3996–11,988	48.93	5.17	1.29	0.94	1.49	0.92	3.48
Total sugar	10	410	FD + SNV	3996–11,988	51.85	5.27	1.32	0.94	1.51	0.92	3.49
Residues	8	410	FD	4759.7–9411.4	48.16	5.34	1.59	0.91	1.68	0.90	3.18
Ratio											
Sug/res	10	425	FD	3996–7197.4, 1185.7–11,988	1.08	0.21	0.07	0.90	0.07	0.88	2.94
Suc/total	13	536	SNV	4242.8–8894.5	0.95	0.03	0.01	0.87	0.01	0.83	2.45
Fru/glc	13	427	FD	4104–7251.4, 8825.1–11,185.7	1.95	0.57	0.17	0.92	0.21	0.87	2.75
Dry weight (online)											
Sugars											
Glucose	19	407	FD	4890.8–11,185.7	0.89	0.49	0.18	0.88	0.21	0.82	2.33
Fructose	12	391	FD	4890.8–7251.4, 8038.3–11,185.7	1.63	0.57	0.27	0.78	0.29	0.74	1.98
Reducing sugar	22	401	FD	4242.8–4767.4, 5785.7–9411.4	2.46	1.02	0.38	0.87	0.44	0.81	2.31
Sucrose	15	514	FD	4890.8–11,972.5	49.52	4.92	1.80	0.87	1.98	0.83	2.49
Total sugar	13	514	FD	4890.8–11,185.7	52.30	5.01	1.84	0.87	1.96	0.85	2.56
Residues	12	495	FD	4890.8–8825.1, 9612–11,972.5	47.52	5.01	1.75	0.88	1.86	0.86	2.69
Ratio											
Sug/res	24	473	FD	4104–11,972.5	1.10	0.20	0.06	0.91	0.08	0.86	2.70
Suc/total	18	396	FD + SNV	4752–6834.8, 7344–9411.4	0.95	0.02	0.01	0.86	0.01	0.81	2.27
Fru/glc	31	396	FD	4104–11,972.5	1.94	0.56	0.18	0.91	0.23	0.83	2.41
Fresh weight (online)											
Moisture	23	528	FD + SNV	4104–11,972.5	73.21	2.46	0.40	0.97	0.46	0.97	5.40
Sugars											
Glucose	34	415	FD	4104–11,972.5	0.25	0.15	0.04	0.93	0.06	0.86	2.66
Fructose	18	405	FD + SNV	4104–11,972.5	0.46	0.18	0.07	0.86	0.08	0.82	2.36
Reducing sugar	24	403	FD	4242.8–5276.6, 5785.7–9411.4	0.67	0.28	0.10	0.88	0.12	0.83	2.39
Sucrose	13	453	FD + SSL	4782.8–11,185.7	13.27	1.91	0.45	0.95	0.49	0.93	3.88
Total sugar	23	514	FD	4104–11,972.5	13.97	2.09	0.50	0.95	0.57	0.93	3.68
Residues	23	459	FD	4104–11,972.5	12.66	1.55	0.43	0.93	0.48	0.90	3.21

N, sample number; SCM, scatter correction methods; SD, standard deviation of reference value; RMSEC, root mean square error of calibration; R^2^, determination coefficient; RMSECV, root mean square err of cross validation; R^2^cv, determination coefficient of cross validation; RPD, ratio performance deviation; SNV, standard normal variate; SSL, straight line subtraction; FD, first derivative; FD + SNV, a combination of FD and SNV; FD + SSL, a combination of FD and SSL

A considerable improvement in predictive capacity was observed in both offline and online NIRS modeling via integrative calibration. The newly generated equations should be applicable for prediction of biomass composition content. The suggested models provide multiple options for related high-throughput screening approaches. Notably, online calibration models can play a significant role, as they are substantially advantageous in high-throughput analysis of large-scale sample sets and offer better prospects for practical applications in the future.

## Conclusions

A total of 628 sugarcane accessions were applied for determination of sugarcane stalk quality and NIRS calibration. Large variations in sugar, moisture, insoluble residues and related parameters were detected among these collections, allowing for systematic offline and online NIRS calibrations. Finally, twenty-five models were generated with high *R*^*2*^, *R*^*2*^*cv*, *R*^*2*^*ev*, and RPD values, exhibiting excellent predictive capacity. In particular, online calibration models, owing to their uniquely inherent advantages in high-throughput detection, showed great prospects for application. Hence, this study provided a high-throughput strategy for large-scale screening of optimal sugarcane varieties and precision breeding.

## Methods

### Sample collection

A total of 628 sugarcane varieties representing a wide variation of sugarcane germoplasm were planted in the Fusui experimental field of Guangxi University, Nanning, following the standard agronomic practices for the region. Sugarcane stalks were collected between November 2018 and March 2019 in five different batches. In each collection date (i.e., 1–5), a different number of varieties were harvested. Collections number 1, 2, 3, 4 and 5 comprised, respectively, 164, 162, 184, 70 and 48 varieties. These collections also represent different growth stages during sugarcane maturity. After removing leaves and tips, six randomly selected stalks of each sugarcane variety were used for online NIRS spectrum scanning and further analysis. In addition, the stalks of forty sugarcane genotypies were collected every 20 days from the jointing stage to the ripening stage for model optimization.

### Near-infrared spectral data collection

#### Online NIRS spectrum scanning

The randomly selected fresh stalks were immediately shredded using DM540 (IRBI Machines and Equipment Ltd, Brazil), blended and transmitted by CPS (Cane presentation system, Bruker Optik GmbH, Germany), and NIRS spectral data were simultaneously collected through the MATRIX-F (Bruker Optik GmbH, Germany) online system. The spectrum acquisition was taken by a full-band scanning mode at wavelengths ranging from 4000 to 10,000 cm^−1^ with 4 cm^−1^ steps at room temperature. The spectral absorbance values were recorded as log1/R, where R is the sample reflectance. The obtained continuous reflectance values were then averaged for further analysis.

#### Offline NIRS spectrum scanning

The shredded fresh sugarcane samples were immediately collected and inactivated at 100 °C for 1 h to denature and deactivate the enzymes, as well as to prevent sugar degradation by microbial. Subsequently, the inactivated samples were dried under 60 °C until no loss of weight. The dried samples were ground over 40 mesh for offline NIRS spectrum data collection and further sugar content analysis. MATRIX-F equipped with a Q413 sensor head was used for contactless offline measurements. The reflectance of each sample was recorded and averaged for further calibration analysis.

### Sugarcane stalk quality determination

Moisture content was determined by a standard loss on drying method [34]. Sugar content (g/g, % dry weight) was analyzed by high-performance anion chromatography (HPAEC) method. Briefly, 0.100 g of ground dry sample was extracted with 40 mL ddH_2_O at 50 °C for 2 h. Additionally, 5.0 mL of lactose (1.0 mg/mL, Aladdin Biochemical Technology Co., Ltd., Shanghai, China) was added as an internal standard. The 50 mL sample was then filtered through 0.22 μm membrane filters for HPAEC detection.

ICS 5000^+^ system (Dionex/Thermo Fisher Scientific, Waltham, MA, USA) equipped with a pulsed amperometric detector (PAD) and Carbopac™ PA1 column (250 mm × 4 mm, 10 μm) was employed for determining soluble sugar in sugarcane. The chromatographic conditions were as following: column temperature was set at 30℃; injection volume was 25 μL; eluent A: ddH_2_O; and eluent B: 500 mmol/L NaOH solution (Merck KGaA, Darmstadt, Germany). An isocratic elution procedure of 60% A and 40% B at the flow rate of 2.0 mL/min was used for chromatographic analysis. The “Carbohydrates standard quad" waveform, as described in Additional file 1: Table S1, was employed for PAD.

For sugar content (g/g, % dry weight) calculation, the standard internal method was used for quantitative analysis. Analytical curves were produced using sucrose, d-glucose, and d-fructose as standards. Simultaneously, lactose was added as the internal standard (The standard chemicals were purchased from Aladdin Biochemical Technology Co., Ltd., Shanghai, China). The peak area ratios between each sugar (glucose, fructose, and sucrose) and the internal standard were calculated and corrected by the standard curves and then applied for its quantitative analysis. Insoluble residues content in sugarcane stalks was calculated by deducting the total soluble sugar from dry biomass. The biomass composition content (g/g, % fresh weight) was calculated based on its dry weight and the moisture content in fresh stalks. Biological triplicates were performed for each sample.

### NIRS calibration

The OPUS spectroscopy software (version 7.8, Bruker Optik GmbH, Germany) was used for data processing and NIRS calibration. To solve the problems associated with overlapping peaks and baseline correction, pretreatments, and wavelength ranges, selection of the raw spectral data was performed before calibration. Several kinds of spectral pretreatment methods were provided in OPUS software, including constant offset elimination (COE), straight-line subtraction (SSL), standard normal variate (SNV), Min–Max normalization (MMN), multiplicative scattering correction (MSC), first derivative (FD), second derivative (SED), combinations of the first derivative and straight-line subtraction (FD + SSL), standard normal variate (FD + SNV), and multiplicative scattering correction (FD + MSC). The NIRS spectra were divided into multiple intervals and then reassembled to obtain the optimal spectral region. A principal component analysis (PCA) was conducted to characterize the structure of the spectral population, and the GH outlier (GH > 3.0) samples were eliminated. Partial least square (PLS) regression was performed to generate calibration equations. Internal cross-validation and external validation were carried out to test the performance of the generated calibration equations. The best equations were selected according to a high coefficient of determination of the calibration/internal cross-validation/external validation (*R*^*2*^/*R*^*2*^*cv*/*R*^*2*^*ev*), low root means square error of calibration/internal cross-validation/external validation (RMSEC/RMSECV/RMSEP), and high ratio performance deviation (RPD) values [37].

## Supplementary Information

**Additional file 1: Table S1.** Waveform used in PAD for HPAEC detecting. **Figure S1.** The comparison of sucrose content in fresh and dried samples. **A**–**B**: sucrose content in fresh and dried sugarcane samples; **CD**: correlation analysis of sucrose content between fresh and dried sugarcane samples; **E**–**F**: residuals of sucrose content between fresh and dried sugarcane samples. ***indicated the significant correlation at p < 0.001 level. **Table S2.** Statistics for equations generated for prediction of biomass components in sugarcane stalks.

## Data Availability

All data generated or analysed during this study are included in this published article (and its additional file informations). The grounded dried sugarcane samples are available at Guangxi Key Laboratory of Sugarcane Biology, Guangxi University, Nanning, Guangxi, China.
