# Supplementary material for: A systematic high-throughput phenotyping assay for sugarcane stalk quality characterization by near-infrared spectroscopy
Source: Plant Methods. 2021 Jul 13;17:76. doi: 10.1186/s13007-021-00777-8 (PMC8278626; doi:10.1186/s13007-021-00777-8)
Supplement: Supplementary file 1 — Additional file 1: Table S1. Waveform used in PAD for HPAEC detecting. Figure S1. The comparison of sucrose content in fresh and dried samples. A–B: sucrose content in fresh and dried sugarcane samples; CD: correlation analysis of sucrose content between fresh and dried sugarcane samples; E–F: residuals of sucrose content between fresh and dried sugarcane samples. ***indicated the significant correlation at p < 0.001 level. Table S2. Statistics for equations generated for prediction of biomass components in sugarcane stalks. [file 13007_2021_777_MOESM1_ESM.pdf]

Annex 1 Waveform used in PAD for HPAEC detecting

**Waveform Time/s**	**Potential/v**	**Ramp**
**0.00**	0.1	
**0.20**	0.1	begin
**0.40**	0.1	end
**0.41~0.42**	~2.0	
**0.43**	0.6	
**0.44~0.5**	~0.1	

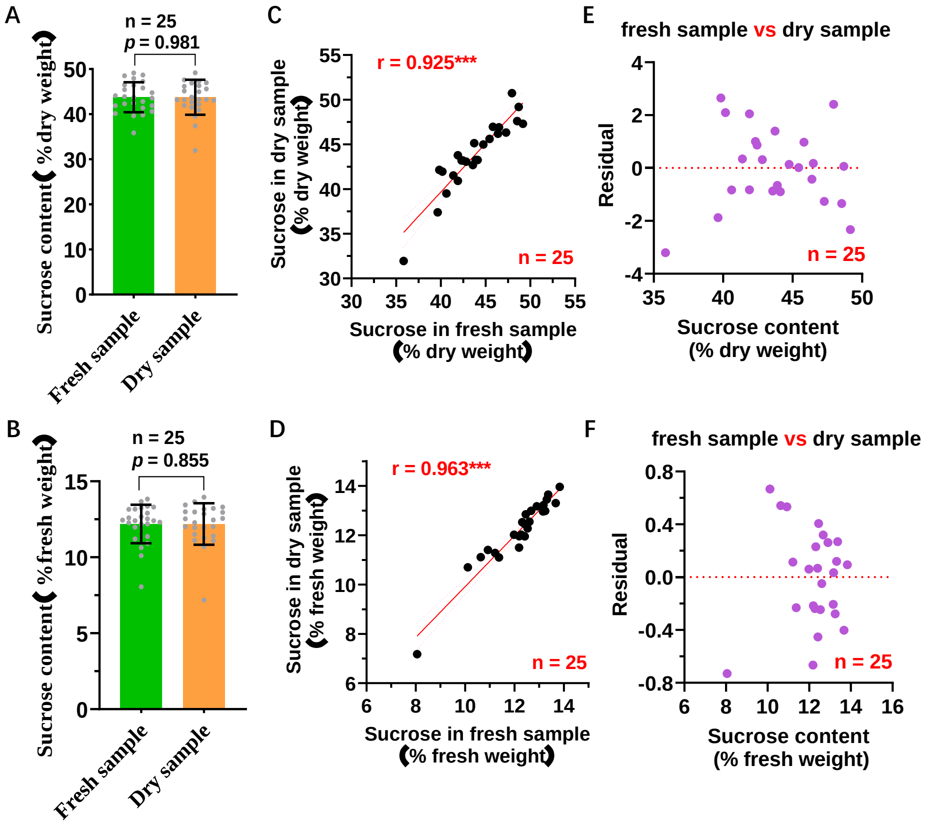

Annex 2 The comparison of sucrose content in fresh and dried samples. A-B: sucrose content in fresh and dried sugarcane samples; **C-D:** correlation analysis of sucrose content between fresh and dried sugarcane samples; **E-F:** residuals of sucrose content between fresh and dried sugarcane samples. *** indicated the significant correlation at $p < 0.001$ level.

Annex 3 Statistics for equations generated for prediction of biomass components in sugarcane stalks.

	Calibration								Cross validation			External validation		
	Rank	N	SCM	Spectrum range (cm^{-1})	Mean	SD	RMSEC	R^2	RMSECV	R^2cv	RPD	N	RMSEP	R^2ev
**Dry weight (Offline)**														
**Sugars (% dry weigh)**														
Glucose	16	415	FD	4104~ 7251.4,8825.1~ 9612	0.90	0.61	0.19	0.91	0.24	0.84	2.52	103	0.23	0.92
Fructose	11	379	FD+SNV	4104~10398.8	1.66	0.69	0.25	0.87	0.31	0.79	2.20	100	0.33	0.87
Reducing sugar	12	399	FD+SNV	4104~7251.4,8825.1~9612,10398.8~11185.7	2.65	1.34	0.42	0.91	0.51	0.86	2.64	106	0.61	0.89
Sucrose	18	396	FD+SNV	4104~ 7251.4,8038.3~ 10398.8	49.25	5.13	1.59	0.91	2.39	0.78	2.14	101	1.87	0.89
Total sugar	6	367	FD	4890.8~ 5677.7,6464.5~ 7251.4,8038.3~ 10398.8	51.82	5.20	1.99	0.86	2.11	0.83	2.46	102	1.91	0.88
**Residues**	10	374	SNV	4242.8~5276.6,5793.4~7344,7860.8~8377.7	48.04	5.18	1.85	0.88	2.02	0.85	2.57	100	1.81	0.89
**Ratio**														
Sug/Res	13	399	FD	3996~11988	1.09	0.21	0.08	0.86	0.10	0.75	2.02	104	0.09	0.82
Suc/Total	14	433	FD	4242.8~7860.8,8886.8~9403.7	0.95	0.03	0.01	0.87	0.01	0.81	2.27	110	0.01	0.89
Fru/Glc	13	388	SNV	4759.7~5276.6,5793.4~9411.4	2.00	0.57	0.22	0.85	0.30	0.72	1.90	96	0.23	0.81
**Dry weight(Online)**														
**Sugars**														
Glucose	16	290	FD	4890.8~11185.7	0.89	0.49	0.19	0.86	0.25	0.74	1.98	117	0.21	0.81
Fructose	20	272	FD	4104~8038.3,8825.1~11972.5	1.60	0.53	0.23	0.83	0.30	0.68	1.76	104	0.27	0.79
Reducing sugar	31	285	FD	4242.8~9411.4	2.44	0.97	0.33	0.90	0.52	0.71	1.86	116	0.46	0.84
Sucrose	10	382	FD+SNV	5677.7~10398.8,11185.7~11972.5	49.59	4.83	1.86	0.86	2.03	0.82	2.38	111	2.35	0.82
Total sugar	8	396	FD+SNV	5677.7~7251.4,8038.3~11972.5	52.31	4.84	1.95	0.84	2.09	0.81	2.31	112	2.36	0.80
**Residues**	13	390	FD	4752~6834.8,7853.1~9411.4	47.40	4.88	1.75	0.88	1.90	0.85	2.56	108	1.99	0.86
**Ratio**														
Sug/Res	22	332	FD	4104~11972.5	1.11	0.20	0.06	0.91	0.08	0.86	2.63	131	0.07	0.88
Suc/Total	19	280	FD	4752~9411.4	0.95	0.02	0.01	0.88	0.01	0.77	2.07	110	0.01	0.81
Fru/Glc	27	282	FD	4242.8~7344,7853.1~9411.4	1.92	0.53	0.18	0.90	0.26	0.77	2.09	114	0.25	0.83
**Fresh weight(Online)**														
**Moisture**	22	456	FD+SNV	4104~ 11972.5	73.24	2.36	0.45	0.97	0.55	0.95	4.30	114	0.63	0.96
**Sugars**														
Glucose	31	295	FD	4104~11972.5	0.25	0.16	0.04	0.93	0.07	0.78	2.13	117	0.05	0.87
Fructose	18	289	FD+SNV	4104~11972.5	0.45	0.18	0.07	0.87	0.08	0.81	2.31	116	0.08	0.82
Reducing sugar	24	287	FD	4242.8~9411.4	0.67	0.28	0.10	0.89	0.13	0.79	2.19	116	0.12	0.81
Sucrose	22	413	FD+MSC	4104~11972.5	13.32	1.93	0.54	0.93	0.67	0.88	2.89	110	0.63	0.92
Total sugar	16	446	FD	5276.6~ 7344,7853.1~ 8377.7	14.05	2.00	0.61	0.91	0.68	0.89	2.96	112	0.62	0.93
**Residues**	11	420	FD+SNV	5276.6~9411.4	12.60	1.47	0.58	0.85	0.62	0.82	2.38	112	0.63	0.88

N, sample number; SCM, scatter correction methods; SD, standard deviation of reference value; RMSEC, root mean square error of calibration; R^2, determination coefficient; RMSECV, root mean square error of cross validation; R^2cv, determination coefficient of cross validation; RMSEP, root mean square error of external validation; R^2ev, determination coefficient of external validation; RPD, ratio performance deviation; SNV, standard normal variate; MSC, standard multiple scatter; FD, first derivative; FD+SNV, a combination of FD and SNV; FD+MSC, a combination of FD and MSC.
